# Mechanical cues regulate *in vitro* implantation of mouse embryos

**DOI:** 10.1016/j.mtbio.2026.103105

**Published:** 2026-04-18

**Authors:** Qian Liu, Kai Chen, Shaoshan Pan, Tao Zhang, Qian Wang, Jiaying Zhu, Jin Zhang, Xiaohua Jiang, Haiying Jia, Shengxia Zheng, Tianzhi Luo

**Affiliations:** a Department of Modern Mechanics, Reproductive Medicine Center, The First Affiliated Hospital, State Key Laboratory of Fire Science, University of Science and Technology of China, Hefei, China; b Jiangsu Hernrrie Biological and Medical Technology Company, Taizhou, China; c The Ninth Medical Center of Chinese People's Liberation Army General Hospital, Beijing, China

**Keywords:** Embryo implantation, Substrate stiffness, Actin cytoskeleton, Traction force, Hydrogel

## Abstract

Uterine mechanical properties, particularly tissue stiffness, are dynamically regulated during the embryo implantation window to ensure optimal mechanocompatibility. However, maternal aging and fibrotic pathologies, such as adenomyosis and intrauterine adhesions, disrupt this balance, leading to increased stromal stiffness due to abnormal extracellular matrix (ECM) deposition and crosslinking. In this study, we simulated various uterine environments *in vitro* by adjusting the mechanical cues, aiming to exploring embryonic mechanosensing and invasive capabilities during implantation. Our observations revealed that embryos cultured on softer substrates demonstrated enhanced developmental efficiency and exhibited distinct assembly morphologies compared to those on stiffer substrates. Using traction force microscopy (TFM), we quantified the traction forces exerted by embryos on soft substrates, noting a gradual increase in these forces before they stabilized within a specific range and displayed sustained fluctuations that were closely linked to cytoskeletal remodeling. We further identified that the traction force provided a real-time assessment of the developmental status of the embryo during implantation. Additionally, given that the mechanosensitive Yes-associated protein (YAP) differentially responds to substrate stiffness, we further investigated its pivotal role during early embryogenesis by RNA-seq. This research offers insights into mechanics-based interventions that could potentially enhance the outcomes in assisted reproductive technologies.

## Graphical abstract

**Figure:**
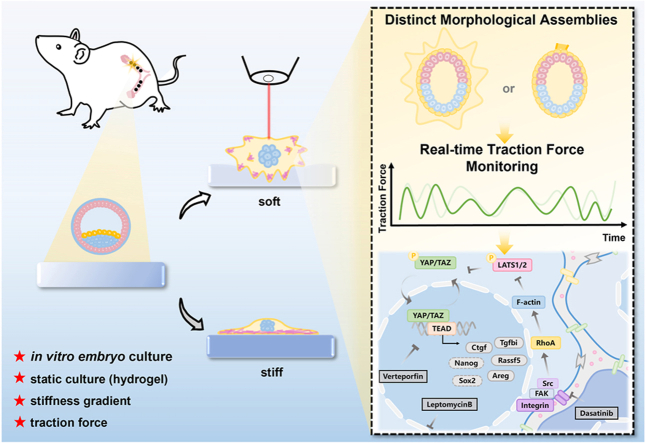

## 1 Introduction

Embryo implantation is a highly coordinated and intricate process that depends on interactions between the developing blastocyst and the receptive endometrium [1,2]. Traditionally, this interaction has been understood to be primarily regulated by a sophisticated network of biochemical signals. This network includes the spatiotemporally specific expression and interplay of ovarian hormones (such as estradiol and progesterone), cytokines, chemokines, and adhesion molecules (such as integrins and cadherins) [3,4]. In mice, the embryos develop into blastocysts by embryonic day (E) 3.5, consisting of the pluripotent epiblast (EPI), trophectoderm (TE), and primitive endoderm (PrE) [5]. Implantation takes place around E4.5, at which point the TE differentiates into polar TE and mural TE [6]. The latter attaches to the uterine epithelium and secretes enzymes such as matrix metalloproteinases (MMPs) to remodel the endometrium, thereby facilitating successful implantation [7]. Beyond biochemical factors, physical and mechanical forces are also essential for regulating cell behavior and tissue morphogenesis [[8], [9], [10]]. Particularly in cases of advanced maternal age or the presence of uterine pathologies, the mechanical properties of the uterus, including stiffness and viscoelasticity, might undergo substantial changes [[11], [12], [13]]. These alterations could complicate the interactions between the embryo and the maternal uterus. Mechanical factors not only impact the physical contact between the embryo and the endometrium but might also regulate embryonic development and implantation via mechanotransduction pathways, thereby exerting critical effects on cellular behavior [14].

The introduction of *in vitro* fertilization (IVF) technology in the 1930s enabled the visualization of embryo implantation and post-implantation development processes [[15], [16], [17]]. This includes traditional static culture [18,19], roller culture systems [20], and circulator systems. Subsequent advances in *in vitro* embryo culture technology have focused primarily on refining the culture environment. This includes the introduction of specific biochemical factors and the optimization of circulatory metabolic systems to better mimic the *in vivo* microenvironment and support the healthy embryonic development [21,22]. These technological improvements have not only enabled *in vitro* recapitulation of embryo-maternal uterine interactions and subsequent developmental processes, but also provided powerful tools for real-time monitoring and precise measurement [19,23,24]. This has greatly advanced research into how various biological and environmental factors affect embryo implantation and post-implantation development [25,26]. Kevin S. Kolahi reported that mouse embryos exhibit sensitivity to mechanical signals from fertilization through the blastocyst stage, with embryos cultured on collagen-coated substrates of 1 kPa stiffness (Col-1kPa) showing higher developmental rates [27]. Zhen Gu successfully supported the development of mouse blastocysts to the early organogenesis stage *in vitro* by mimicking the mechanical properties and microstructure of the mouse uterus using PDMS and collagen [28]. These studies, utilizing hydrogels to simulate uterine mechanical properties *in vitro*, demonstrate that early embryonic development is profoundly influenced by external mechanical cues. However, most of these studies focus on optimizing the *in vitro* developmental environment rather than investigating the dynamic processes of implantation and post-implantation development itself [29,30].

Cells anchor to the ECM via adhesion molecules such as integrins [31,32]. By contracting their actomyosin cytoskeleton, they exert traction forces on the ECM, driving processes including cell migration, morphological changes, and tissue remodeling [[33], [34], [35]]. Successful embryo implantation relies on continuously evolving interactions between the blastocyst and the endometrium [29,36,37]. Within this dynamic framework, the traction forces actively generated by embryonic trophoblast cells are crucial effectors that drive adhesion and invasion. Traditional research, relying heavily on endpoint histological analyses, could reveal gene expression patterns or receptor localization at specific time points [19,38]. However, this approach inherently fails to capture the transient mechanical events that occur during this dynamic interplay. This limitation significantly hampers our understanding of the mechanisms of implantation and struggles to explain why embryos with ‘normal’ morphological and molecular characteristics may still face implantation failures. Traction force microscopy (TFM)—combining high spatiotemporal resolution imaging with biomechanical computational models—offers a solution to this challenge [39,40]. Elucidating how embryo-derived traction forces dynamically regulate the physical interactions and bidirectional signaling at the embryo-endometrium interface is of great significance for deepening our understanding of the mechanical regulation of implantation [9,29]. Despite its potential, research exploring this mechanical dimension remains relatively scarce, thereby limiting our comprehensive understanding of the pathological mechanisms underlying reproductive disorders such as recurrent implantation failure (RIF) and early pregnancy loss.

In this study, we established *in vitro* culture environments with varying stiffness (1 ∼50 kPa) using GelMA gels to observe the implantation stage of mouse embryos. Based on observed morphological developmental differences, we have specifically designed experiments to investigate the underlying structural dynamics governing embryonic development on soft substrates. Our experimental approach focused on quantifying the spatiotemporal patterns of traction forces generated during implantation and their association with embryonic migration. To dissect the potential molecular mechanisms governing these forces, we employed targeted perturbations directed at key mechanosensitive components, including actin polymerization, myosin II contractility, and ROCK and FAK signaling pathways. Furthermore, we investigated the potential of traction force mapping as a novel approach for the non-invasive, and *in situ* assessment of embryonic remodeling dynamics. Finally, recognizing the central role of mechanotransduction, our experimental design incorporated an investigation into the activity of the core mechanosensitive transcriptional regulator YAP, while also examining potential alterations in gene expression induced by the dysregulation of YAP signaling pathway. This study aims to provide novel biomechanical insights and potential intervention targets to improve the success rates of assisted reproductive technologies (ART).

## 2 Methods

### 2.1 Animal and ethical approval

ICR 8-week-old females and 10-week-old males were purchased from SPF (Suzhou) Biotechnology. All animal experiments were conducted in accordance with the Guide for the Care and Use of Animals for Research Purposes. All the procedures were approved by the Institutional Animal Care and Use Committee at the First Affiliated Hospital of USTC (code no. 2025-NA-0123).

### 2.2 Preparation of GelMA gels

Two distinct GelMA gels (GelMA30; GelMA90, EFL) were used in the fabrication of substrates with four different stiffness values. Four hydrogel formulations were prepared by dissolving GelMA in PBS with 0.1% w/v LAP: 5% GelMA30 (∼1 kPa), 10% GelMA30 (∼4 kPa), 15% GelMA30 (∼10 kPa), and 15% GelMA90 (∼50 kPa). GelMA solutions were incubated in a 50 °C water bath until complete dissolution of the GelMA solid and disappearance of foam. To ensure sterility, filter the GelMA solutions through a 0.22 μm pore size filter (SLGPR33RB, Millipore Express).

Petri dishes and 25 mm glass coverslips were treated to be either hydrophilic or hydrophobic, serving as substrates for mouse embryo culture. To minimize the impact of hydrogel thickness on subsequent experiments, we typically used 40 μL of hydrogel to maintain a thickness of approximately 150 μm. The hydrogel was exposed to ultraviolet light (LS1601, EFL) to ensure adequate cross-linking. After preparation, the hydrogel was washed with PBS, followed by the addition of culture medium.

To prepare hydrogel substrates embedded with fluorescent microspheres (F8813, Invitrogen) for traction force measurement, we divided the hydrogel into two layers during substrate preparing. The lower layer provided the required substrate stiffness, while the upper layer was loaded with fluorescent microspheres. The microspheres were diluted in hydrogel to prepare the upper layer (1:100). Approximately 35 μL of hydrogel was used for the lower layer, and about 2 μL for the upper layer to ensure a monolayer distribution of the fluorescent microspheres. After preparation, confocal microscopy was used to check to ensure uniform distribution of fluorescent microspheres. Throughout the preparation and subsequent use, exposure to light was minimized to avoid photobleaching.

### 2.3 Mouse embryo *in vitro* culture

A 100 μL of PMSG solution (100 IU/mL, M2630, Nanjing Aibei) was injected into each female ICR mouse. After 42-48 h, 100 μL of the HCG solution (100 IU/mL, M2530, Nanjing Aibei) was administered, and the female mice were then mated with wild-type male mice. Pregnant mice were humanely euthanized at 3.5 days post-coitum through cervical dislocation, and the embryos were flushed out with prewarmed M2 medium (M1250, Nanjing Aibei) [18]. Under a dissecting microscope (MZ62, Mshot), a group of 10-15 embryos was transferred using a mouth pipette into AT solution (T1788, Sigma-Aldrich) to remove zona. Blastocysts were transferred into prewarmed culture medium covered with mineral oil (M8410, Sigma-Aldrich). IVC1 medium for embryos on days 0-2: CMRL 1066 (11530037, Invitrogen) + 1 × penicillin-streptomycin (60162ES76, YEASEN) + 1 × GlutaMAX Supplement (35050061, Thermo) + 1 × MEM Non-Essential Amino Acids Solution (C3240, Vivacell) + 0.5 × N-2 Supplement (17502048, Gibco) + 0.5 × B-27 Supplement (17504044, Gibco) + 20% FBS (2206371, Bioexplorer). IVC2 medium for embryos on day 3: CMRL 1066 + 1 × penicillin-streptomycin +1 × GlutaMAX Supplement +1 × MEM Non-Essential Amino Acids Solution +30% KnockOut Serum Replacement (10828028, Gibco) [28]. Embryos were cultured at 37 °C in a humidified atmosphere containing 5% CO_2_.

### 2.4 Drug treatment

During the embryo culture experiments, various inhibitors were employed for treatment. Cytoskeletal inhibitors included: Cytochalasin D (10 μM, MedChemExpress) for 30 min, Blebbistatin (50 μM, Abcam) for 30 min, Y-27632 (30 μM, Selleck) for 30 min, and PF-573228 (10 μM, HY-10461, MedChemExpress) for 30 min. For these cytoskeletal inhibitor treatments, additional observations of YAP (expression/localization) changes were conducted at 30 min and 2 h post-treatment. YAP inhibitors—Leptomycin B (100 ng/mL, Selleck), Verteporfin (1 μM, Sigma), and Dasatinib (10 μM, MedChemExpress)—were added at 36 h of embryonic culture and maintained for 12 h before proceeding to the next experimental step. For observing embryonic morphological development on day 3 of culture, the aforementioned inhibitors were added at 48 h of embryonic culture and maintained for 24 h before subsequent experiments. To exclude solvent-induced effects, dimethyl sulfoxide (DMSO; A1458605, Ambeed) was added to the culture medium of control groups at a final concentration consistent with that in inhibitor-treated groups.

### 2.5 Immunofluorescence staining and morphological classification scheme

Embryos were fixed about 20 min with 4% PFA (G1101, Servicebio) and permeabilized for 1 h with 1% Triton X-100 (9002-93-1, BBI Life Sciences) in PBS at 37 °C. Subsequently, embryos were blocked for 1 h in blocking buffer (PR30008, Proteintech) at 37 °C or overnight at 4 °C. For immunostaining, embryos were incubated with primary antibodies at 4 °C overnight. The primary antibodies included OCT4 (1:100, C30A3, Cell Signaling Technology), CDX2 (1:100, 3038-1-AP, Proteintech), E-cadherin (1:100, 20874-1-AP, Proteintech), p-MLC (1:100, 3675T, Cell Signaling Technology), YAP (1:100, sc-101199, Santa Cruz), Vinculin (A2752, Abclonal) and CoraLite Plus 488‐conjugated Phalloidin (PF00001, Proteintech). The following secondary antibodies were incubated for 2 h at room temperature: CoraLite488-conjugated goat anti-rabbit (1:500, SA00013-2, Proteintech), AbFluor 594 goat anti-rabbit (1:500, RF608, Immunoway), Alexa 594 goat anti-mouse (1:500, AB0162, Abways), Alexa Fluor 647 goat anti-rabbit (1:500, A21244, Invitrogen), and AbFluor 647 goat anti-mouse (1:500, RS23420, Immunoway). For nuclear staining, embryos were incubated with 10 μg/mL Hoechst 33342 (CM03169, Proteintech) in PBS at RT for 10 min. The embryos were imaged on a confocal microscope (TCS SP8, Leica).

To quantify the morphological differences in embryo assemblies on day 3 of *in vitro* culture, we performed immunofluorescence staining for F-actin and E-cadherin. In the typical “sun-like” morphology, a core structure (yellow) surrounded by a continuous and outwardly spreading cell layer (red) could be observed at a specific focal plane, resembling the sun. In contrast, in the traditional assembly morphology, the core structure detached from the basal focal plane, and under the same imaging conditions, only this main body could be identified, while the surrounding spreading cell layer could not be fully observed. In this study, we defined the three-dimensional aggregate composed of OCT4-positive epiblast cells and CDX2-positive trophoblast cells with a diameter greater than 80 μm as the “core structure” for morphological quantitative analysis [38].

### 2.6 Nanoindentation

Nanoindentation was performed using Piuma Nanoindenter from Optics 11 Life (Netherlands). Cantilevers with a stiffness of 0.016 N/m and spherical tips with a radius of 3.000 μm were employed for measurements. The Young's modulus was derived from force vs. indentation curves using a Hertzian contact model. A Poisson's ratio of 0.5 was applied, following convention for hydrated biological tissues. For each sample group, a minimum of 10 individual measurement points were acquired at randomly selected locations on the surface of embryos to ensure spatial representation and minimize positional bias. The mean value of these measurements was calculated to obtain representative elastic modulus values for comparison across different sample groups. During data analysis, any data points exhibiting irregular force-distance curves—indicating improper tip-sample contact during indentation (e.g., sliding, adhesion artifacts, or surface contamination)—were systematically excluded from analysis to preserve data integrity.

### 2.7 Time-lapse imaging and migration assay

For long-term monitoring of embryonic development, a live cell workstation (LHSW-GJJ-A, Luohua) was used, which maintained an optimal environment at 37 °C with 5% CO_2_ in a humidified atmosphere. The fluorescence inverted microscope (IX73, Olympus) is equipped with software programmed to capture images at regular intervals tailored to the specific experiment requirements. This setup ensures precise recording and analysis of embryonic developmental stages over the culture period. Embryo morphology was analyzed in FIJI image analysis software.

For the embryo migration assay, embryo migration behavior was also analyzed using FIJI. It was necessary to register and correct the drift during the imaging process, and then measure the displacement of the embryo along the X and Y axes. Time-lapse images of embryos were acquired every 20 min for 24 h. The movement trajectory, average migration speed, directionality ratio and mean square displacements (MSDs) during embryonic development were calculated [[41], [42], [43]].

### 2.8 Traction force measurement

A two-layer hydrogel substrate was fabricated for traction force microscopy [44]. The bottom layer (approximately 20 μL) provides the desired substrate stiffness. Subsequently, a thin top layer containing fluorescent beads (F8813, Invitrogen) was formed by applying only 2 μL of hydrogel, which was pre-mixed with beads at a 100:1 (v/v) hydrogel-to-bead ratio, and flattening it with a coverslip (25 mm). This resulted in a single, well-distributed plane of fiducial markers for displacement tracking. After preparing hydrogel substrates embedded with fluorescent microspheres, culture oil was omitted from the dish to facilitate subsequent experiments. Instead, an increased volume of medium above 500 μL was used to prevent excessive evaporation. Each dish was inoculated with approximately 10 ∼ 15 blastocysts and cultured at 37 °C, 5% CO_2_. Given that laser confocal microscopy requires a certain amount of scanning time, inoculating excessive embryos could disrupt the scheduling of subsequent imaging intervals.

Fluorescent bead images were acquired using a laser scanning confocal microscope (TCS SP8, Leica) equipped with a 20 × objective lens (NA 0.75). The fluorescence signal was detected by a photomultiplier tube (PMT) to generate high-contrast images with a digital resolution of 1024 × 1024 pixels. To assess embryo adhesion and traction forces generated during development, continuous imaging of embryos was performed for up to 4 h at 5-min intervals under optimal culture conditions using a live cell imaging workstation. In some experiments, actin was labeled using fluorescent dyes (CY-sc001, Cytoskeleton) at concentrations of 0.1 nM for siRNA and 10 μM for verapamil, with staining performed for 10 h. For the cytoskeletal inhibitor experiments, initial imaging was conducted for 10 min without inhibitor at 5-min intervals. Following inhibitor addition, imaging was continued for 30 min, with the interval between shots reduced to 2 min. For YAP inhibitor experiments, inhibitors were added at 36 h of culture and maintained until 48 h, after which traction force imaging was conducted. Displacement fields describing hydrogel deformation were determined by analyzing fluorescent microsphere images before and after removing adhering embryos via treatment with 1 μM NaOH (10019764, Sinopharm Chemical Reagent).

Microscope stage drift during multi-position imaging can introduce significant displacement in the acquired time-series data, compromising the accuracy of traction force calculations. To correct for this instrumentation-induced displacement of fluorescent beads, experimental drift was compensated using the Template Matching plugin in FIJI. The traction force field was reconstructed by using an optimal filtering approach based on fourier transform traction cytometry (FTTC). All calculations and image processing were performed in MATLAB [45].

### 2.9 Nuclear to cytoplasmic YAP ratio

The nuclear-to-cytoplasmic (Nuc/Cyto) YAP ratio was quantified using FIJI software and subsequently analyzed with Microsoft Excel [46,47]. The nuclear area was delineated using Hoechst 33342 staining, while the cellular area was defined by the actin cytoskeleton, which was visualized via Phalloidin staining. Nuclear and cellular outlines were stored in the FIJI ROI (Region of Interest) manager, which facilitated the quantification of integrated density for both the cell (YAP_Cell_) and the nucleus (YAP_Nuc_) in the YAP channel. Therefore, YAP_Nuc/Cyto_ was calculated as follows:

$${\text{YAP}}_{\text{Cyto}}={\text{YAP}}_{\text{Cell}}\;–\;{\text{YAP}}_{\text{Nuc}}$$

$${\text{YAP}}_{\text{Nuc}/\text{Cyto}}={\text{YAP}}_{\text{Nuc}}\;/\;{\text{YAP}}_{\text{Cyto}}$$

### 2.10 RNA-seq library preparation and data analysis

Total RNA was isolated from dissected whole mouse embryo tissues using the TRIzol® method, followed by DNase I digestion to eliminate genomic DNA contamination. RNA integrity was assessed using an Agilent 2100 Bioanalyzer (RNA Integrity Number, RIN ≥8.0), and quantity was verified with a Qubit2.0 fluorometer (total RNA ≥100 ng). Following library preparation, initial library quantification was performed with a featureCounts. Libraries were diluted to 1.5 ng/μL and insert size distribution was assessed using an Agilent 2100 Bioanalyzer. Paired-end sequencing (150 bp) was conducted on the Illumina NovaSeq 6000 platform employing sequencing by synthesis (SBS) chemistry.

Bioinformatic analysis followed a standard pipeline: High-quality reads were aligned to the *Mus musculus* reference genome (GRCm38/mm10) using HISAT2. Gene expression quantification was performed using the featureCounts within the Subread package. Differential expression analysis was performed with DESeq2, applying thresholds of |log_2_FC| > 1 and FDR <0.05 to identify significantly differentially expressed genes. Gene Ontology (GO) was conducted using ClusterProfiler, with a significance threshold of padj <0.05. Protein interaction networks for differentially expressed genes were generated using STRING database interactions, with network analysis conducted through the DIAMOND pipeline.

### 2.11 Statistical analyses

Data were analyzed using Bonferroni post hoc test or two-tailed Student's t-test. Figure legends indicate total sample sizes (n) pooled across all experimental runs. Each experimental group was tested in ≥3 independent experiments, with multiple samples assessed per run. All quantitative data were expressed as the mean ± standard error of means (means ± SEM). Statistical significance was defined as *p < 0.05, **p < 0.01, and ***p < 0.001. Non-significant differences are denoted by the abbreviation “ns”.

## 3 Results

### 3.1 Improved development of mouse embryos cultured *in vitro* on soft substrates

**Fig. 1:**
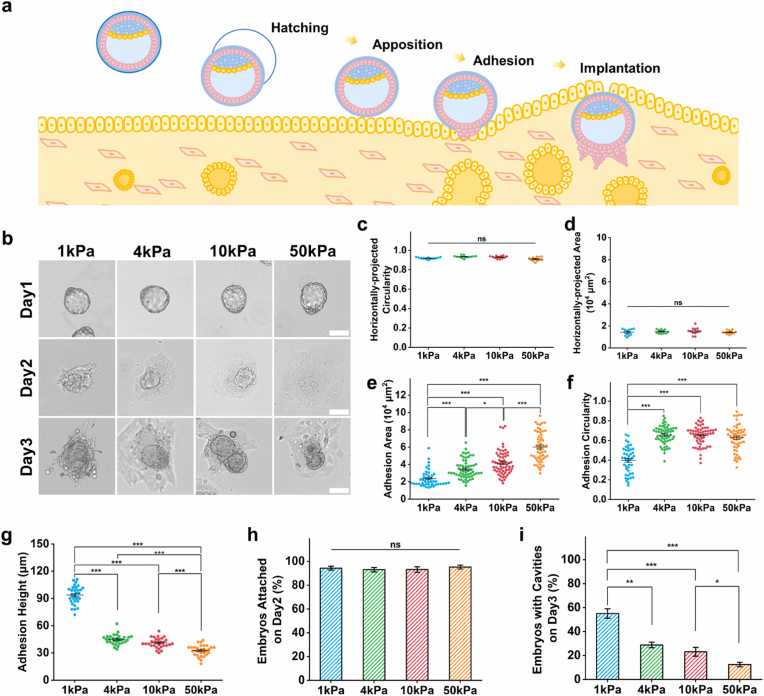
Morphological characterization of mouse embryos cultured *in vitro* on substrates with different stiffness levels. **(a)** Schematic diagram illustrating the process of mouse blastocyst implantation. **(b)** Representative brightfield images of mouse embryos cultured *in vitro* on a 1 kPa, 4 kPa, 10 kPa, and 50 kPa substrates at each time period. Scale bar, 100 μm. **(c)** Quantification of horizontally-projected circularity of embryos on IVC day 1. 1 kPa (n = 12), 4 kPa (n = 12), 10 kPa (n = 12), and 50 kPa (n = 12). **(d)** Quantification of horizontally-projected area (10^4^ μm^2^) of embryos on IVC day 1 of embryos. 1 kPa (n = 12), 4 kPa (n = 12), 10 kPa (n = 12), and 50 kPa (n = 12). **(e)** Quantification of adhesion circularity of embryos on IVC day 2. 1 kPa (n = 44), 4 kPa (n = 57), 10 kPa (n = 58), and 50 kPa (n = 55). **(f)** Quantification of adhesion area (10^4^ μm^2^) of embryos on IVC day 2. 1 kPa (n = 44), 4 kPa (n = 59), 10 kPa (n = 61), and 50 kPa (n = 53). **(g)** Quantification of adhesion height (μm) of embryos on IVC day 2. 1 kPa (n = 30), 4 kPa (n = 30), 10 kPa (n = 30), and 50 kPa (n = 30). **(h)** Percentage of attached embryos on IVC day 2. n = 177, 186, 173, and 150 for the 1 kPa, 4 kPa, 10 kPa, and 50 kPa groups, respectively. **(i)** Percentage of embryos with cavities on IVC day 3. n = 116, 111, 129, and 100 for the 1 kPa, 4 kPa, 10 kPa, and 50 kPa groups, respectively. One-way ANOVA with Bonferroni post hoc test was used for statistical analysis. *P < 0.05, **P < 0.01, ***P < 0.001.

In studies of embryo development, maternal uterine pathologies are often accompanied by fibrotic processes. Alterations in uterine mechanical properties—resulting from ECM deposition and cross-linking—may represent key biomechanical factors contributing to implantation failure or pregnancy complications. To further investigate the dynamic developmental processes of embryo adhesion and implantation within the uterine context (Fig. 1a), an *in vitro* culture model that allows real-time visualization is critical. Here, we obtained fresh blastocysts from wild-type mice (Fig. S1) and cultured them on GelMA gels with different stiffness levels to evaluate embryonic growth and development [48].

The stiffness range employed in this study was derived from reported values of human uterine tissue under both physiological and pathological conditions [13,49]. Although the mouse model may not fully replicate these absolute stiffness values, the elastic modulus of the mouse uterine horn is also on the kilopascal (kPa) scale [28]. Importantly, the range we established comprehensively encompasses the full biomechanical spectrum from normal to severely compromised tissue states [50,51]. Four distinct substrate stiffnesses were tested: 1 kPa, 4 kPa, 10 kPa, and 50 kPa. Mouse embryos were cultured *in vitro* on these substrates (Fig. 1b), with their adhesion to the substrate and early developmental progression monitored throughout the culture period. To quantify these dynamic processes, we statistically analyzed the developmental morphology of the embryos. On day 1 of *in vitro* culture (IVC day 1), most embryos had not yet adhered to the substrate. At this stage, we quantified two key morphological parameters: the projected circularity and projected area of the embryos. No significant differences were observed between the groups at this initial time point (Fig. 1c and d). On IVC day 2, embryos cultured on substrates with different stiffness exhibited distinct morphological changes. Consistent with observations in cell culture, the adhesion area of the embryos increased with rising substrate stiffness (Fig. 1e). However, compared to embryos on stiffer substrates, those cultured on the 1 kPa substrate displayed significantly lower circularity (Fig. 1f) and greater height (Fig. 1g). Embryos on soft substrates also began to demonstrate distinct growth morphologies: well-developing embryos were fully adhered to the substrate by this time, while non-adherent embryos remained non-adherent even with prolonged culture. This finding indicated that substrate stiffness is not the critical factor determining whether an embryo adheres to the substrate (Fig. 1h). On IVC day 3, more developed embryos began to form a characteristic epiblast (EPI) cavity—a key structural marker we used to assess embryonic development on this day. Statistical analysis showed that the developmental rate—defined as the percentage of embryos forming an EPI cavity—approached 60% for embryos on soft substrates, compared to less than 20% for those on the 50 kPa substrate (Fig. 1i). This result demonstrated that mouse embryos cultured *in vitro* on softer substrates exhibit superior developmental outcomes.

### 3.2 Embryos assemble into unique developmental morphology on soft substrates

**Fig. 2:**
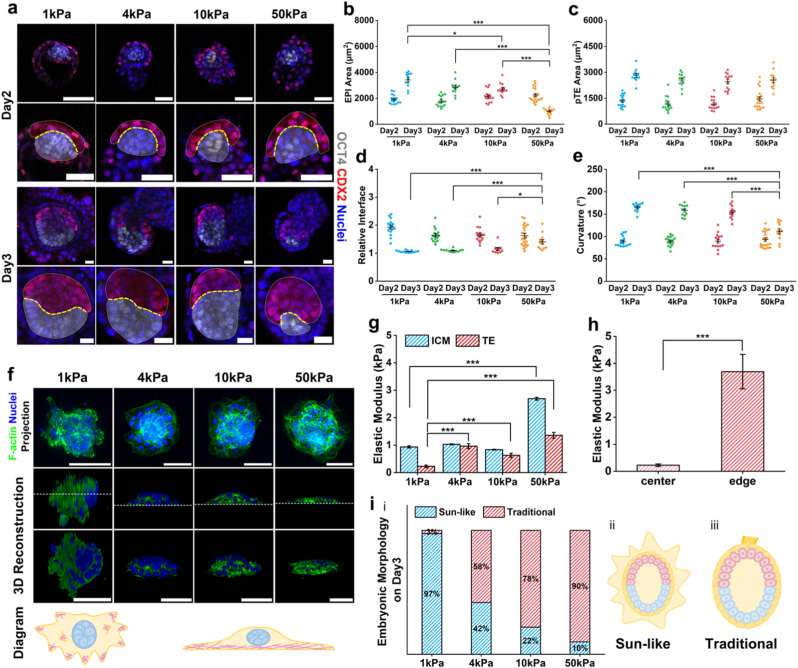
**Embryos exhibit unique morphological patterns on soft substrates. (a)** Representative images of embryonic morphology on IVC day 2 and day 3. Top row: embryos stained with OCT4 (white), CDX2 (red), and nuclei (blue). Bottom row: zoom-in views of the EPI and pTE lineages, with pTE highlighted in red, the EPI in white and the interface in yellow. Scale bars: 50 μm (Day2), 20 μm (Day3). **(b)** Quantification and comparison of EPI area (μm^2^) on IVC day 2 and day 3. The 1 kPa group was colored blue, the 4 kPa colored group green, the 10 kPa group colored red, and the 50 kPa group colored orange. **(c)** Quantification and comparison of pTE area (μm^2^) on IVC day 2 and day 3. **(d)** Quantification and comparison of relative interface on IVC day 2 and day 3. **(e)** Quantification and comparison of curvature (°) on IVC day 2 and day 3. Day2: 1 kPa (n = 16), 4 kPa (n = 16), 10 kPa (n = 15), 50 kPa (n = 20). Day3: 1 kPa (n = 12), 4 kPa (n = 12), 10 kPa (n = 13), 50 kPa (n = 22). Scatterplot, mean ± SEM. **(f)** Embryos stained with F-actin (green), and nuclei (blue) on IVC day 2. Top panel: max intensity projections of the embryos. Middle panel: side views after 3D reconstruction. Bottom panel: images after tilting the side views 45°. Schematic illustrations of morphological differences are shown. Scale bar, 100 μm. **(g)** The elastic modulus (kPa) of embryos at different substrate stiffness levels was measured by nanoindentation. ICM: 1 kPa (n = 11), 4 kPa (n = 21), 10 kPa (n = 24), 50 kPa (n = 10). TE: 1 kPa (n = 22), 4 kPa (n = 10), 10 kPa (n = 12), 50 kPa (n = 40). Histogram, mean ± SEM. **(h)** The elastic modulus (kPa) of embryos cultured on 1 kPa substrate was measured by nanoindentation. Distribution of cell regions relative to the inner cell mass (ICM), with the proximal region labeled as the center (n = 22) and the distal region as the edge (n = 19). Histogram, mean ± SEM. **(i)** (i)The proportion of embryos exhibiting various morphologies across different substrate stiffness levels. 1 kPa (n = 78), 4 kPa (n = 48), 10 kPa (n = 41), and 50 kPa (n = 30). (ii-iii) schematic illustration of morphological differences. Statistical analysis was performed using a two-tailed Student's t-test. *P < 0.05, **P < 0.01, ***P < 0.001. (For interpretation of the references to color in this figure legend, the reader is referred to the Web version of this article.)

The transition from the pre-implantation to post-implantation state in embryonic development lays the foundation for subsequent morphogenesis. This transition could be characterized by changes in the embryo's tissue shape and topological architecture. Our previous experiments revealed that mouse embryos cultured *in vitro* on soft substrates already exhibited distinct morphological alterations on IVC day 2, a phenomenon warranting further observation. During *in vivo* morphogenesis, the mouse embryo undergoes characteristic changes: its epiblast (EPI) area and polar trophectoderm (pTE) area gradually increase, the relative interface approaches 1, and the interface curvature increases [38]. To more clearly characterize the dynamic remodeling process by which the epiblast transforms from an elliptical shape to a cup-like structure under different substrate stiffnesses *in vitro*, we performed immunofluorescence staining for OCT4 (marking EPI) and CDX2 (marking pTE) on embryos at IVC day 2 and day 3 (Fig. 2a). The experimental results showed that there were no significant differences in epiblast development between groups cultured on substrates with different stiffnesses on IVC day 2. However, on IVC day 3, embryos cultured on soft substrates exhibited a significantly larger EPI area (Fig. 2b), a relative interface closer to 1 (Fig. 2d), and higher interface curvature (Fig. 2e) compared to those on stiff substrates. In contrast, no significant differences in pTE area were observed between the groups at this stage (Fig. 2c). Furthermore, analysis of EPI height (Fig. S2a), EPI width (Fig. S2b), pTE height (Fig. S2c), interface length (Fig. S2d), and interface diameter (Fig. S2e) revealed that embryos cultured on soft substrates consistently exhibited morphological parameters that more closely resemble physiologically relevant developmental states than those on stiff substrates. These findings indicated that embryos cultured on soft substrates remodel more rapidly into the tissue architecture that is characteristic of the implantation-competent state.

The alteration of cellular mechanical state is closely linked to the dynamic reorganization of the cytoskeleton [52]. Accordingly, we performed staining for the embryonic actin cytoskeleton and reconstructed its architecture in 3D (Fig. 2f) to analyze changes in overall structural organization and spatial patterning. We discovered that mouse embryos cultured on 1 kPa substrates exhibited prominent accumulation of actin specifically at the regions that interact with the substrate, and the substrate itself showed noticeable deformation (Fig. S3a). Under other stiffness conditions, actin distribution was more uniform. We supplemented this observation by staining for actin and myosin II (Phospho-Myosin Light Chain 2,pMLC). The result showed that on 1 kPa substrates, myosin II also accumulated predominantly at regions interacting with the substrate and showed significant co-localization with actin (Fig. S3b). To further investigate actin distribution, the nanoindentation technique was employed to measure the elastic modulus of different regions within embryos (Fig. S4a). We divided embryos into two main parts: the Inner Cell Mass (ICM), which represents the core developmental compartment, and the Trophectoderm (TE), which provides essential developmental conditions (Fig. S4b). Measurements revealed that the TE exhibited a lower elastic modulus than the ICM (Fig. 2g), suggesting that under the given substrate stiffness conditions, the TE likely provides a more suitable microenvironment for ICM growth. For embryos cultured on 1 kPa substrates, we further subdivided the TE of these embryos into the central region (center, adjacent to the ICM) and the peripheral region (edge, distal to the ICM) to investigate the specific distribution of actin. The elastic modulus of the central region was approximately 0.22 kPa, while the peripheral region exhibited a significantly higher modulus about 3.69 kPa—a difference of nearly 20-fold (Fig. 2h). These findings correlated well with the observed actin accumulation in the 3D reconstructions. We proposed that, in response to external mechanical cues on soft substrates, the embryos remodel more effectively into an implantation-competent state. The higher elastic modulus in the TE periphery might facilitate substrate invasion, while the softer central region adjacent to the ICM likely provides a more compliant environment that is conducive to further development of the core tissues.

As culture progressed, we observed that embryos on substrates with different stiffness levels primarily assembled into two distinct morphological types (Fig. S5). One type resembled a more conventional *in vitro* developmental state, where the main embryonic body detached from the basal layer and the substrate surface [28,29]. The other type, which we termed the “Sun-like” morphology, featured the main embryonic body tightly enveloped by the outer cells. Statistical analysis of the developmental morphologies across different stiffnesses revealed that embryos on soft substrates predominantly adopted the Sun-like morphology, while those on stiff substrates more frequently formed the conventional state (Fig. 2i). Strikingly, 97% of embryos on 1 kPa substrates assembled into the Sun-like morphology. Given our prior finding that soft substrate promotes earlier development of the EPI cavity, we proposed that the soft substrate conditions *in vitro* drive the assembly of an implantation-suitable tissue architecture (the Sun-like morphology), which in turn facilitates further growth and development.

### 3.3 Enhanced development of the epiblast for embryos on soft substrates

**Fig. 3:**
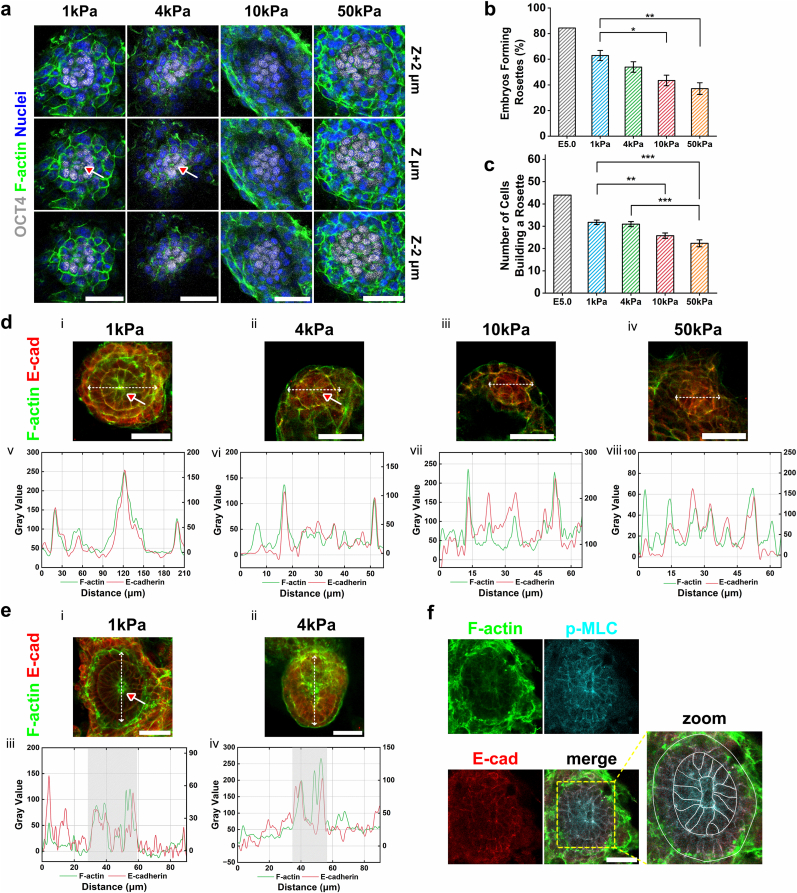
Improved epiblast development in embryos on soft substrates. **(a)** Embryos stained with the EPI marker OCT4 (white), F-actin (green) and nuclei (blue). Z-slice images were arranged in order from low to high, with a 2 μm interval. Red arrows indicate the core of rosettes. Scale bar, 50 μm. **(b)** Percentage of embryos forming rosettes on IVC day 2. n = 108, 78, 80, and 90 for 1 kPa, 4 kPa, 10 kPa, and 50 kPa, respectively. **(c)** Number of cells per rosette. n = 36, 28, 18, and 23 for 1 kPa, 4 kPa, 10 kPa, and 50 kPa, respectively. **(d)** Top panel (i-iv): representative images of EPI on IVC day 2. The embryos stained with F-actin (green) and E-cadherin (red). Red arrows highlight regions of colocalization between F-actin and E-cadherin. Scale bar, 100 μm. Bottom panel (v-viii): merged plot profiles of the EPI, from 1 kPa to 50 kPa. **(e)** Top panel (i-ii): representative images of EPI on IVC day 3. Embryos stained with F-actin (green) and E-cadherin (red). Red arrows indicate the EPI cavity. Scale bar, 100 μm. Bottom panel (iii-iv): merged plot profiles of the EPI, with a gray background to emphasize the location of the EPI cavity. **(f)** Representative images of epiblast on day 2 of *in vitro* culture. The embryos stained with F-actin (green), phospho-Myosin Light Chain 2 (cyan) and E-cadherin (red). Statistical analysis was performed using a two-tailed Student's t-test. *P < 0.05, **P < 0.01, ***P < 0.001. (For interpretation of the references to color in this figure legend, the reader is referred to the Web version of this article.)

During intrauterine development and hatching, the epiblast transforms from a relatively simple spherical structure into rosettes composed of polarized cells as it progresses from the pre-implantation to post-implantation stage. Between E4.5 and E5.0, an increasing percentage of embryos assemble these rosettes—a structure critical for normal development and serving as the foundation for the nascent neural egg cylinder [53]. To assess rosette formation under different substrate stiffness *in vitro*, we performed OCT4 and F-actin co-staining on IVC day 2 embryos and conducted multi-plane imaging to visualize structural assembly (Fig. 3a). Experiments revealed that the percentage of embryos forming rosettes was significantly higher on soft substrates compared to those on stiff ones. Notably, over 60% of embryos on 1 kPa substrates exhibited rosette formation (Fig. 3b). As development advanced, the number of cells incorporated into rosettes also increased. Consistent with these observations, embryos cultured on soft substrates had a substantially higher total number of cells assembled into rosettes within the epiblast compared to those on stiff substrates (Fig. 3c). These results indicated that epiblasts cultured on soft substrates assemble implantation-competent architecture more rapidly, whereas those on stiff substrates undergo more pronounced developmental delay.

During the transition of epiblast cells into a cup-shaped morphology, tissue remodeling is driven not only by actomyosin-mediated contractile forces at the single-cell level but also through the mechanical integration and propagation of intercellular forces [38]. E-cadherin, expressed from early embryonic stages, forms cell-cell junctions that facilitate tension transmission across the tissue and functions as a mechanosensory module [54]. To investigate force heterogeneity during epiblast remodeling on substrate with different stiffnesses, we performed co-staining of F-actin and E-cadherin. On IVC day 2, all substrate groups exhibited partial colocalization of F-actin and E-cadherin. However, the 1 kPa group showed pronounced co-accumulation of both proteins in the epiblast center (Fig. 3d). Subsequent triple staining (F-actin/myosin II/E-cadherin) confirmed enhanced co-clustering of all three components specifically on soft substrates (Fig. S6a). This indicated elevated contractile force generation in the central epiblast at this stage, potentially initiating EPI cavity formation. To further verify whether actomyosin plays an essential role in rosettes assembly, we treated embryos with assembled rosette structures with inhibitors (Cytochalasin D, CytoD, an actin polymerization inhibitor; Blebbistatin, BLEB, a non-muscle myosin II inhibitor). The results showed that while the structure did not immediately disappear, it became loosely assembled (Fig. S6e). On IVC day 3, the 1 kPa group maintained central colocalization of F-actin and E-cadherin alongside cavity formation, supporting force transmission (Fig. 3e, Fig. S6b). Myosin was also observed at cavitation sites, indicating sustained contractility (Fig. 3f, Fig. S6c). In contrast, embryos on stiff substrates exhibited disrupted colocalization patterns, implying impaired force propagation that correlates with disorganized morphogenesis and compromised developmental competence. We further compared embryos with two distinct assembly morphologies cultured on 1 kPa substrates (Fig. S7a). Although EPI cavity formation in both types was accompanied by actin and E-cadherin co-localization, the Sun-like embryos formed significantly larger cavities (Fig. S7b). This increased cavity size suggests that the Sun-like architecture may be associated with a more advanced developmental outcome. These results demonstrated that optimal epiblast remodeling occurs on soft substrates (1 kPa), where contractile forces that may exist within the rosette could promote EPI cavity formation (Fig. S6d). Successful morphogenesis critically depends not only on the coordinated generation of forces within the tissue, but also on their mechanical transmission.

### 3.4 Dynamic traction force generation in embryos on soft substrates

Understanding traction force generation during embryo-substrate interactions is crucial for elucidating embryo implantation. To observe this process, the approximate timing of embryonic adhesion must first be determined [42]. We conducted 24-h time-lapse imaging of embryos (Fig. S8a), quantifying adhesion area and circularity (Fig. S8b). Results revealed significant inflection points in area-circularity plots at 32 h post-seeding across all groups, with stabilization occurring after 36 h, indicating the initiation and stabilization of embryonic adhesion. Consequently, traction force measurements were initiated at 32 h post-seeding. To evaluate potential inaccuracies in traction force reconstruction caused by substantial MMP-2/MMP-9-induced alterations in substrate stiffness [55,56], we performed *in situ* measurement of the stiffness of the matrix immediately surrounding the embryo. We found that the stiffness remained stable at its initial value of ∼1 kPa (Fig. S9), indicating that the level of MMP secretion by the embryos was insufficient to alter the mechanical properties of the substrate.

**Fig. 4:**
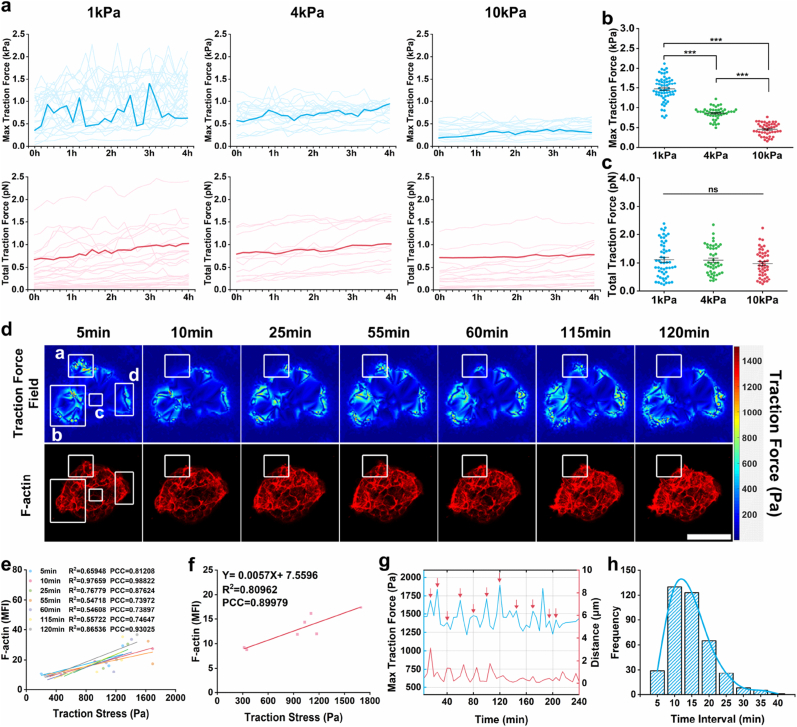
Dynamic traction force in embryos on soft substrates. **(a)** Curves of max traction force (Pa, top panel) and total traction force (nN, bottom panel) over time. Dark blue shows representative data, while light blue indicates data from multiple replicates. 1 kPa (n = 26), 4 kPa (n = 20), and 10 kPa (n = 18). **(b)** Quantification of max traction force (Pa) of embryos on substrates with different stiffness levels. 1 kPa (n = 61), 4 kPa (n = 46), and 10 kPa (n = 45). **(c)** Quantification of total traction force (nN) of embryos on substrates with different stiffness levels. 1 kPa (n = 53), 4 kPa (n = 45), and 10 kPa (n = 45). **(d)** Top panel: representative images of the traction force field. Bottom panel: corresponding images labeled for F-actin, aligned with the traction force field images. Four representative ROIs (a, b, c, and d) in the four images correspond to the same ROIs used to calculate F-actin MFI and traction stress strengths. Scale bar, 100 μm. **(e)** Correlation between traction stress strength (Pa) and F-actin MFI across different regions. **(f)** Correlation between traction stress strength (Pa) and F-actin MFI at different time points. **(g)** Correlation analysis of max traction force (Pa) and displacement (μm) during embryonic development on 1 kPa substrates. The max traction force is indicated by the blue line, while the displacement is represented by the red line. **(h)** Frequency analysis of periodic max traction force. Data were from 29 embryos across 4 independent experiments. Statistical analysis was performed using a two-tailed Student's t-test. *P < 0.05, **P < 0.01, ***P < 0.001. (For interpretation of the references to color in this figure legend, the reader is referred to the Web version of this article.)

Mouse embryos were placed on GelMA gels embedded with fluorescent microspheres. Lateral hydrogel deformation—reflecting traction forces exerted by embryos on the substrate—was measured and calibrated by tracking microsphere displacements. Confocal microscopy recorded time-lapse sequences of embryos and microspheres to characterize spatiotemporal dynamics of traction forces over 4 h at 5-min intervals. Traction forces were measured on substrates with stiffness of 1 kPa, 4 kPa, and 10 kPa, given the superior embryonic development observed on soft substrates. Peak traction force was defined as the max traction force (Pa) generated by individual embryos, while total traction force (nN) was calculated by integrating average traction with embryo-substrate contact area [44]. Results demonstrated that during initial substrate interactions, both max and total traction forces progressively increased across all groups (Fig. S10, with 1 kPa data shown as a representative example). Subsequently, max traction force ceased continuous growth and exhibited fluctuating variations within a defined range, while total traction force gradually stabilized (Fig. 4a). Increasing substrate stiffness significantly reduced max traction force (Fig. 4b), whereas total traction force remained statistically unchanged (Fig. 4c). This indicated that embryos exert finite mechanical output, but dynamically redistribute traction forces in response to environmental stiffness. The 1 kPa substrate was selected for subsequent experiments due to its higher max traction force sensitivity, superior force detection resolution, and optimal embryonic developmental outcomes.

To investigate the impact of cytoskeletal remodeling on traction force generation during embryo-substrate interactions, we employed traction force microscopy to analyze the co-distribution of F-actin and traction forces within embryos. We dynamically monitored the cytoskeleton in real-time using live-cell dyes, allowing for prolonged observation of both cytoskeletal organization and traction force distribution (Fig. 4d). Utilizing these datasets, we examined the spatiotemporal dynamics of F-actin and traction forces through linear correlation analysis and Pearson correlation coefficient (PCC) calculations. We first analyzed spatial variations in F-actin mean fluorescence intensity (MFI) and max traction force (Pa) magnitude across different regions within individual embryos. Correlation analysis revealed a consistently correlation between local F-actin MFI and traction force magnitude at various timepoints within the same embryo (Fig. 4e). Subsequent temporal analysis of the same spatial regions demonstrated a linear correlation between F-actin MFI and traction force fluctuations over time (Fig. 4f), confirming that F-actin distribution positively correlates with traction force magnitude in developing embryos.

When the total traction force generated by embryos on substrates plateaued—indicating potential stabilization of embryonic adhesion—max traction force continued to exhibit substantial fluctuations. We hypothesized that this phenomenon might arise from persistent embryonic motility. Time-lapse imaging and migration trajectory analysis throughout embryonic development revealed no substrate stiffness-dependent effects on migration patterns but confirmed continuous embryonic movement (Fig. S 11). We therefore quantified the relationship between embryonic displacement and max traction force fluctuations at 5-min intervals. Our analysis demonstrated that each peak in max traction force was consistently followed by significant displacement within the subsequent 5-min window (Fig. 4g). Arrows highlight these displacement events immediately following traction force maxima. This suggested that large fluctuations in traction forces likely reflect the embryo's dynamic migration process, establishing traction force monitoring as a potential indicator of embryonic motility. Furthermore, recurrent traction force peaks were not stochastic events. Statistical analysis of peak occurrence intervals revealed highly dynamic yet approximately 10-min cyclical patterns (Fig. 4h), confirming periodicity in both embryonic migration and traction force generation. These core findings—real-time observations of max and total traction forces during adhesion and development (Fig. S 12a-b), and the traction force-displacement correlation (Fig. S 12c-e)—were consistently replicated on 4 kPa substrates.

### 3.5 Sources of traction forces during embryo adhesion on soft substrates and potential applications

**Fig. 5:**
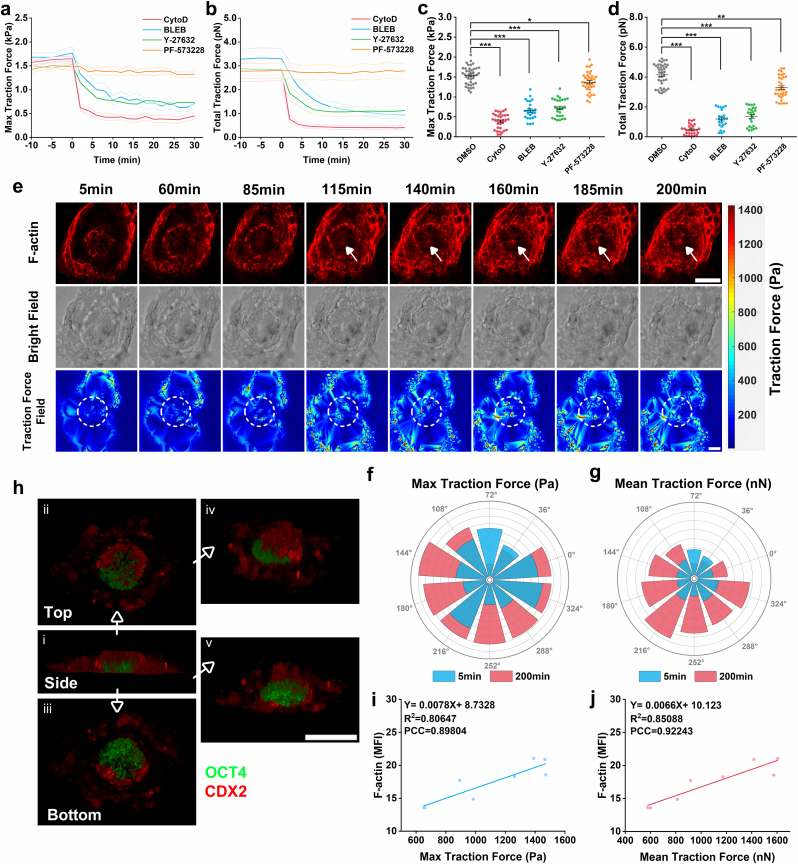
Sources of traction force during embryo adhesion on 1 kPa substrates and their potential applications. **(a)** Curves of max traction force (Pa) of embryos over time after drug administration. CytoD (n = 12), BLEB (n = 8), Y-27632 (n = 10), and PF-573228 (n = 12). Line graph, mean ± SEM. **(b)** Curves of total traction force (nN) of embryos over time after drug administration. Gray background was used to mark the time period before drug administration. CytoD (n = 12), BLEB (n = 8), Y-27632 (n = 9), and PF-573228 (n = 11). Line graph, mean ± SEM. **(c)** Quantification of max traction force (Pa) of embryos treated with different inhibitors. DMSO (n = 40), CytoD (n = 33), BLEB (n = 25), Y-27632 (n = 26), and PF-573228 (n = 37). Scatterplot, mean ± SEM. **(d)** Quantification of total traction force (nN) of embryos treated with different inhibitors. DMSO (n = 39), CytoD (n = 28), BLEB (n = 23), Y-27632 (n = 25), and PF-573228 (n = 34). Scatterplot, mean ± SEM. **(e)** Top panel: corresponding images labeled for F-actin, aligned with the traction force field images. Middle panel: bright field images. Bottom panel: traction force field images. White arrows indicate the core of rosettes. Scale bar, 50 μm. **(f)** The graphs illustrate the max traction forces (Pa) at different positions within the circular annotated region. Key time points are highlighted: the 5-min is indicated in blue, and the 200-min is indicated in red. **(g)** The graphs illustrate the mean traction forces (nN) at different positions within the circular annotated region. **(h)** Multi-view images after three-dimensional reconstruction. Embryos stained with the EPI marker OCT4 (green) and extraembryonic ectoderm marker CDX2 (red). (i) Side view of the 3D reconstruction of stained images. (ii) top-down view of the 3D reconstruction of stained images. (iii) bottom-up view of the 3D reconstruction of stained images. (iv) top-down view rotated 30° towards the upper left. (v) Side view rotated upwards by 45°. Scale bar, 100 μm. **(i)** Correlation between max traction force (Pa) and F-actin MFI. **(j)** Correlation between mean traction force (nN) and F-actin MFI. Statistical analysis was performed using a two-tailed Student's t-test. *P < 0.05, **P < 0.01, ***P < 0.001. (For interpretation of the references to color in this figure legend, the reader is referred to the Web version of this article.)

Dynamic monitoring of traction forces enabled visualization of force interactions between embryos and substrates, subsequently prompting us to investigate the molecular mechanisms underlying traction force generation. Our primary targets were the cytoskeleton and its associated proteins, including actin, myosin II, ROCK, and FAK [57,58]. To examine their roles in traction force generation, we used dimethyl sulfoxide (DMSO) as a control and employed specific inhibitors: Cytochalasin D, Blebbistatin, Y-27632 (a Rho kinase inhibitor), and PF-573228 (a FAK inhibitor) to disrupt the function of these proteins [59,60]. To more clearly delineate the impact of these proteins on traction force generation, we performed real-time traction force observations on individual embryo before and after inhibitor treatment. Experimental results demonstrated a rapid decline in both max (Fig. 5a) and total traction forces (Fig. 5b) following inhibitor treatment, with the Cytochalasin D-treated group exhibiting the fastest decrease. Statistical analysis of the final max (Fig. 5c) and total traction forces (Fig. 5d) post-inhibition revealed reductions across all four treatment groups: the Cytochalasin D group reached minimal levels, while the Blebbistatin and Y-27632 groups exhibited intermediate reductions. These findings indicated that actin, myosin II, ROCK, and FAK collectively contribute to traction force generation during embryo-substrate interactions, with actin exerting the most significant influence.

During prolonged imaging of embryonic development, we observed the gradual formation of rosettes via actin cytoskeleton labeling, with the centers of these rosettes marked by white arrows (Fig. 5e). As rosettes became progressively apparent, the traction force field—initially concentrated only at the embryonic periphery—began to show detectable forces in the central region. Analysis of traction force images at 5 and 200 min revealed significant increases in both max (Fig. 5f) and mean traction forces (Fig. 5g) within the central area. Given that embryos cultured on 1 kPa substrates displayed greater height, whereas two-dimensional traction force measurements primarily detect forces at the cell-substrate interface, we investigated whether central traction forces originated from rosette assembly. Embryos were co-stained with OCT4 and CDX2, followed by Z-stack scanning and imaging (Fig. S13). 3D reconstruction confirmed that EPI assembly indeed occurred at the basal region interacting with the substrate (Fig. 5h). In contrast, under other stiffness conditions, embryos predominantly adopted a conventional architecture where the developing embryonic body detached from basal cells and the substrate surface, preventing observation of this phenomenon. To further validate the correlation, we performed linear regression and PCC analyses between the F-actin MFI of rosettes and max/mean traction force (Fig. 5i and j). Results demonstrated a positive correlation between F-actin MFI and both max and mean traction forces. The max traction forces were largely associated with the pushing forces of the invasion pseudopodium of the extravillous trophoblasts at the embryo-substrate interface, driving the invasion of the embryo into the substrate. The soft substrate allows the invasion to progress without spending much mechanical power whereas the stiff substrate impedes the invasion. It is highly possible that the invasion of the outer trophoblasts effectively pulls the inner EPI cells and triggers the formation of rosettes, a critical milestone for subsequent maturation in embryonic development [53]. Traction force measurement technology provides a non-invasive approach to monitor the embryo's physical state and its interactions with the external environment by analyzing traction forces exerted on the substrate.

### 3.6 Impact of the mechanosensitive protein Yes-associated protein (YAP) on the *in vitro* development of mouse embryos

**Fig. 6:**
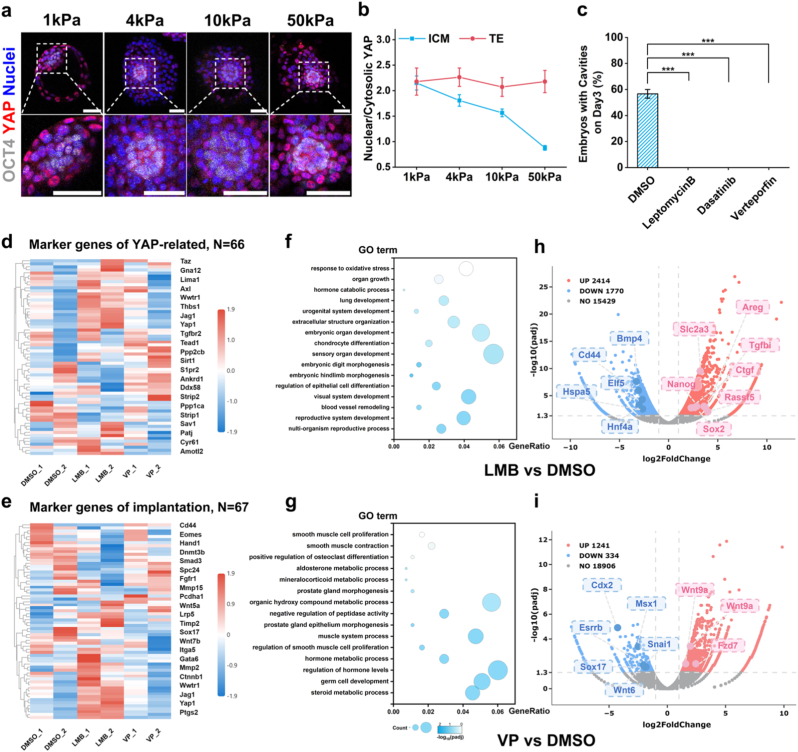
**YAP dysregulation impacts preimplantation embryogenesis *in vitro*. (a**) Representative images showing YAP subcellular localization in embryos cultured on substrates with different stiffness levels on IVC day 2. The DMSO treated group served as the control, while the experimental groups included treatment with Leptomycin B, Dasatinib, and Verteporfin. **(b)** Quantification of the nuclear/cytosolic YAP ratio. n = 25, 22, 22, and 22 for 1 kPa, 4 kPa, 10 kPa, and 50 kPa, respectively. **(c)** Percentage of cavitated embryos following treatment with YAP-related inhibitors on IVC day 3 (n = 45). **(d**–**e)** Scaled expression heatmap of YAP signaling and embryonic implantation marker genes, based on hierarchical clustering of RNA-seq data. The x-axis represents sample names. The y-axis represents the FPKM-normalized values of differentially expressed genes. Red intensity indicates higher expression levels, while blue intensity indicates lower expression levels. **(f)** GO enrichment analysis of Leptomycin B-treated embryos versus controls (DMSO). Bubble chart displays the significant GO terms selected from the enrichment results. The x-axis represents the gene ratio, and the y-axis represents GO terms. GO terms with padj <0.05 were considered significantly enriched. **(g)** GO enrichment analysis of Verteporfin-treated embryos versus controls (DMSO). **(h)** Volcano plot displaying differential gene expression following Leptomycin B treatment versus control embryos. Data points are plotted according to log_2_FoldChange on the x-axis and -log_10_(padj) on the y-axis. Gray dashed lines indicate significance thresholds; red points represent upregulated genes and blue points represent downregulated genes. **(i)** Volcano plot displaying differential gene expression following Verteporfin treatment versus control embryos. Statistical analysis was performed using a two-tailed Student's t-test. *P < 0.05, **P < 0.01, ***P < 0.001. (For interpretation of the references to color in this figure legend, the reader is referred to the Web version of this article.)

Yes-associated protein (YAP) is a key mechanotransducer in mammalian development, dynamically shuttling between the cytoplasm and nucleus in response to ECM stiffness [61,62]. The TE relies on YAP-mediated mechanosensing to coordinate embryonic adhesion and invasion while the EPI utilizes nuclear YAP to maintain pluripotency [63,64]. We examined YAP localization in embryos cultured *in vitro* on substrates with different stiffness levels on IVC day 2 (Fig. 6a). Notably, YAP was consistently localized in the nuclei of TE cells. Conversely, EPI cells exhibited a substrate stiffness-dependent nucleocytoplasmic distribution of YAP, with a predominantly nuclear localization on soft substrates and a cytoplasmic one on stiff substrates (Fig. 6b). Furthermore, embryos cultured on stiff substrates exhibited noticeable developmental delay. This suggested that maintaining the YAP localization profile on soft substrates supports normal embryonic development and sustains post-implantation pluripotency. To further investigate the critical role of YAP in development, we treated embryos *in vitro* with several YAP inhibitors: Leptomycin B (LMB, a nuclear export protein inhibitor), Dasatinib (an SRC-family tyrosine kinase inhibitor), and Verteporfin (VP, a TEAD-YAP interaction disruptor) [65]. The results indicated that although embryos treated with inhibitors remained stably adherent to the substrate (Fig. S14a), they failed to sustain normal development (Fig. 6c). Additionally, we quantified the traction forces exerted between the inhibitor-treated embryos and the substrates. Analysis of the traction force maps did not reveal significant changes in max or total traction forces (Fig. S14b–c), but did show variations in force distribution (Fig. S14d). Moreover, continuous monitoring under conditions of sustained YAP activation or inhibition indicated that the periodicity of traction force peaks remained largely unchanged (Fig. S14e). Given our previous analysis of traction force generation, the interplay between YAP signaling and force distribution might involve other key players in YAP related feedback loop.

To identify these potential players, we performed RNA-Seq to examine the transcriptomic signatures induced by Leptomycin B and Verteporfin treatment and conducted unsupervised hierarchical clustering of the significantly differentially expressed genes (DEGs) (|log_2_FC| > 1, padj <0.05). DEGs revealed clear segregation between treatment groups (LMB, VP) and controls (Fig. S15a), confirming robust transcriptomic reprogramming. To dissect the functional drivers of this divergence, we focused on gene sets representing critical biological processes perturbed by these treatments. Transcriptional analysis of the YAP-related gene subset revealed significant differences between inhibitor-treated and control embryos (Fig. 6d), confirming effective inhibitor intervention at the gene expression profile level. Subsequent clustering analyses of gene subsets related to embryo implantation (Fig. 6e), hematopoiesis, and early organogenesis (Fig. S15b) further indicated that YAP/TAZ inhibition broadly affects multiple key developmental processes. This section incorporated results from GO enrichment analysis, which revealed significant differences in biological processes including organ growth, cell proliferation, embryonic organ development, and metabolic processes (Fig. 6f and g). To transcend granular gene-level descriptions and capture the spectrum of transcriptomic alterations, volcano plots were generated. Leptomycin B-treated embryos exhibited upregulation of pluripotency factors (*Nanog*, *Sox2*) and the glucose transporter *Slc2a3*, with concomitant downregulation of developmental regulators *Bmp4*, *Cd44*, *Elf5*, *Hspa5*, and *Hnf4a*. YAP/TAZ signaling targets (*Ctgf*, *Tgfbi*, *Rassf5*, *Areg*) were potently induced (Fig. 6h). YAP inhibition resulted in the downregulation of key genes regulating cell differentiation, including the trophectoderm transcription factor *Cdx2*, the definitive endoderm marker *Sox17*, the pluripotency-associated nuclear receptor *Esrrb*, and the epithelial-mesenchymal transition regulator *Snail*. Expression of *Msx1*, a gene critical for embryo implantation, was also significantly reduced. In contrast, expression of the upstream pathway components *Fzd7*, a Wnt receptor, and *Wnt9a*, a Wnt ligand, was notably increased. This upregulation likely represents a cellular feedback mechanism attempting to counteract YAP inhibition and restore pathway homeostasis (Fig. 6i). Transcriptomic analysis revealed marked dysregulation of embryo implantation-related processes following treatment with YAP-related inhibitors which impaired the normal developmental progression of embryos. Notably, the utility of Leptomycin B as a CRM1 inhibitor for enforcing YAP nuclear retention and subsequent activation is constrained by its non-specific mechanis m. Unlike Verteporfin's precise disruption of the YAP-TEAD binding interface, LMB's broad inhibition of CRM1 targets compromises pathway specificity.

## 4 Discussion

Diseases such as endometriosis and adenomyosis are accompanied by significant alterations in the mechanical properties of the endometrium, including increased tissue stiffness, loss of elasticity, or heightened heterogeneity of the local mechanical microenvironment, which directly leads to embryo implantation failure and early developmental arrest [66]. To visually investigate embryo implantation and post-implantation development *in vitro*, we utilized hydrogels with tunable mechanical properties to mimic the uterine environment and cultured mouse embryos within this system. We found that embryos cultured on softer substrates not only exhibited higher developmental efficiency but also formed a distinct morphological structure. The actin cytoskeleton was predominantly localized to the outer trophectoderm (TE) region, with the outer TE region exhibiting a higher elastic modulus and inducing significant substrate deformation. Conversely, the region adjacent to the epiblast exhibited a much lower elastic modulus, providing a softer microenvironment for embryonic development. While this architectural phenotype potentially creates a biomechanically favorable microenvironment for morphogenesis, systematic dissection of these integrated regulatory networks remains imperative. We proposed that the soft substrate may provide a lower elastic modulus environment through trophoblast cells, causes the rearrangement of the EPI cells and led to the rosette formation. At this stage, coordinated mechanical forces drive the shift from individual to collective cellular behavior, facilitating EPI cavity formation and establishing the biomechanical basis for subsequent morphogenesis.

During embryo implantation, trophoblast cells initiate biomechanical crosstalk with the endometrial epithelium through force-mediated interactions [67]. To further investigate the mechanical interactions between the embryo and the substrate during implantation, we employed TFM for continuous mechanical monitoring at nanonewton (nN) resolution and minute-level temporal intervals. Our analysis delineated the biomechanical progression governing embryonic adhesion: during initial adhesion establishment, we observed progressive amplification of both max and total traction forces, signifying the progressive maturation of force-transducing interactions between the embryo and substrate. Crucially, upon transition to the stabilization phase, exhibited rhythmic oscillations that were spatiotemporally coupled with embryonic migration trajectories, similar to the pulsed oscillation found in morphogenesis of *Drosophila* and *Caenorhabitis elegans* embryos [68,69]. In fact, this rhythmic traction force mechanistically depends on the mechanobiological feed-back loop consisting of actin polymerization, myosin II contractility, and the regulatory functions of ROCK and FAK. The period of the oscillations relies on the turnover rates of these proteins. Additionally, TFM captured the formation of rosette structures during embryonic tissue remodeling in real time, providing new perspectives for examining embryonic development while allowing observation and evaluation of embryonic physiological and mechanical states. A key limitation is the dimensional mismatch between the 3D nature of rosette assembly in the embryo and the 2D projection-based measurements of traction forces, which requires resolution in future research. Moreover, although this study effectively characterizes embryonic behaviors in controlled mechanical environments, it cannot replicate the highly dynamic mechanical properties of the native extracellular matrix. This represents a common and inherent limitation of current traction force measurement methods based on static parameters.

**Fig. 7:**
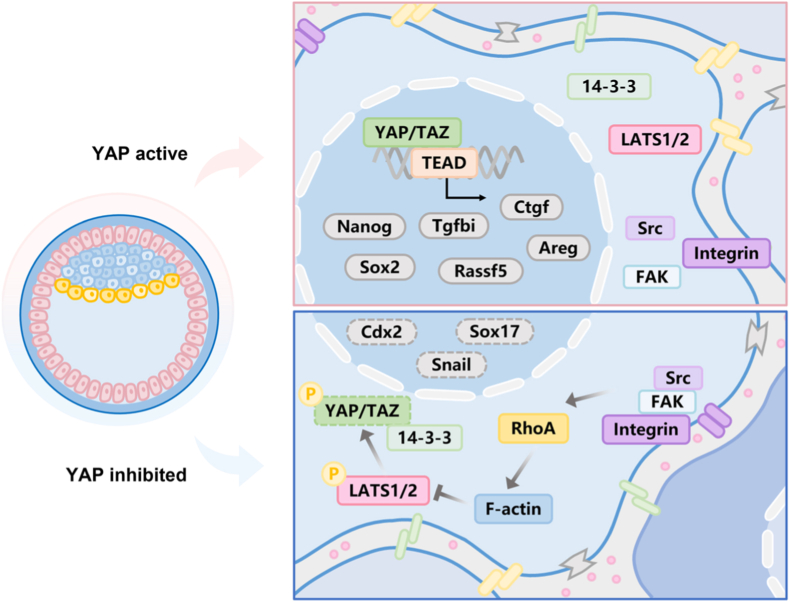
**Regulation of gene expression by YAP in the Hippo pathway during embryo implantation.** Schematic of YAP-mediated gene regulation in the Hippo pathway during embryo implantation. (Top) Hippo pathway inhibition results in constitutive nuclear localization and activation of YAP, driving expression of target genes such as *Nanog*, *Sox2*, *Ctgf*, *Tgfbi*, *Rassf5*, and *Areg*. Experimentally, this activated state can be induced using the nuclear export inhibitor Leptomycin B to sequester YAP in the nucleus. (Bottom) Pharmacological disruption of the YAP-TEAD complex by Verteporfin suppresses YAP transcriptional activity, leading to downregulation of YAP/TEAD-dependent genes including *Cdx2*, *Sox17*, and *Snail*.

YAP is a highly conserved central effector of the Hippo pathway in mammals, regulating the expression of a range of target genes that govern cell fate determination, proliferation, and extracellular matrix remodeling [70]. Thereby, it also plays an indispensable role in critical processes during embryonic development. Before the morula stage, YAP is constitutively active and primarily nuclear-localized, driving the expression of downstream genes essential for blastocoel formation and cell differentiation [61]. In later stages, YAP further contributes to tissue morphogenesis, including processes such as neural formation [71,72] and heart development [73]. This study demonstrates that substrate stiffness regulates YAP distribution, and that aberrant YAP impairs early mouse development by dysregulating gene networks involved in implantation, morphogenesis, and metabolism, ultimately leading to developmental arrest. Based on a YAP functional perturbation screen, we identified a series of genes associated with pluripotency maintenance in stem cells (*Nanog*, *Bmp4*, *Cdx2*, *Sox2*, *Sox17*, etc.). However, the expression levels of *Nanog* and *Sox2* showed no significant difference compared to the control group when YAP was inhibited after the blastocyst stage, a finding consistent with observations in YAP knockdown human embryonic stem cells (hESCs) [74]. Concurrently, we detected significant changes in the expression of genes involved in ECM remodeling and cell migration: connective tissue growth factor (*Ctgf*), angiomotin-like 2 (*Amotl2*), transforming growth factor beta induced (*Tgfbi*), amphiregulin (*Areg*), and CD44 molecule. Further mechanistic exploration of these targets will help reconstruct the dynamic regulatory landscape of YAP in embryonic development (Fig. 7) [75,76]. To further investigate the changes in the spatial distribution of traction forces upon YAP perturbation, we examined biological processes related to the cytoskeleton and extracellular matrix through GO analysis (Fig. S16a-b). Following inhibitor treatment, cytoskeleton-related processes closely linked to traction force generation did not show significant alterations, which aligns with our experimental finding that YAP perturbation does not affect traction force magnitude. However, alterations were observed in key contractility regulators like actin (*Actb*, *Actg1*), and myosin (*Myh9*, *Myh10*, *Myl12b*), implying that YAP perturbation likely exerts its effects not only on dynamic assembly of actin filaments and contractile units, but also targeting other nodes of the cellular mechanosensing machinery such as focal adhesion complex (Fig. S16c). In the context of ECM-related biological processes, we identified a subset of associated genes, including those encoding collagen (*Col1a1*, *Col1a2*, *Col4a1*), integrin (*Itga5*, *Itgb1*), and focal adhesion proteins (*Pxn*, *Zyx*) (Fig. S16d). The specific mechanisms underlying spatial variations in traction forces may warrant further investigation in the context of these genes.

We acknowledge that our current *in vitro* culture model, which incorporates stiffness gradients, lacks the sophistication required to fully capture the impact of substrate stiffness on *in vivo* embryo implantation. The developmental environment provided by the maternal uterine compartment exhibits anisotropy and persistent dynamism [77], while the single static stiffness condition presents certain limitations in replicating developmental requirements. Moreover, although the mouse is a classic model for studying embryo implantation, differences in ECM composition of the endometrium and the molecular basis of embryo-maternal crosstalk compared to humans should be considered when extrapolating the mechanical mechanisms to humans. Furthermore, the biological mechanisms through which mechanical cues regulate this process require further in-depth investigation. Nonetheless, our findings establish a novel mechanobiological framework that provides a new paradigm for understanding force-dependent regulation during both pre- and post-implantation developmental stages.

## Ethics approval and consent to participate

The endometrial sample collection was approved by the Ethics Committee of the First.

Affiliated Hospital of the University of Science and Technology of China (USTC) (2021KY015) and conducted in accordance with the Declaration of Helsinki. All participants gave written informed consent. The animal experiments have been approved from the Institutional Animal Care and Use Committee at the First Affiliated hospital of USTC (code no. 2025-NA-0123).

## CRediT authorship contribution statement

**Qian Liu:** Data curation, Investigation, Methodology, Validation, Visualization, Writing – original draft. **Kai Chen:** Data curation, Methodology. **Shaoshan Pan:** Data curation, Methodology. **Tao Zhang:** Data curation. **Qian Wang:** Data curation. **Jiaying Zhu:** Data curation. **Jin Zhang:** Data curation. **Xiaohua Jiang:** Supervision, Writing – review & editing. **Haiying Jia:** Supervision, Writing – review & editing. **Shengxia Zheng:** Supervision, Writing – review & editing. **Tianzhi Luo:** Conceptualization, Funding acquisition, Writing – review & editing.

## Declaration of competing interest

The authors declare that they have no known competing financial interests or personal relationships that could have appeared to influence the work reported in this paper.

## Data Availability

Data will be made available on request.
