# Supplementary material for: Mechanical cues regulate in vitro implantation of mouse embryos
Source: Mater Today Bio. 2026 Apr 18;38:103105. doi: 10.1016/j.mtbio.2026.103105 (PMC13123377; doi:10.1016/j.mtbio.2026.103105)
Supplement: Multimedia component 1 [file mmc1.docx]

**Mechanical Cues Regulate *In Vitro* Implantation of Mouse Embryos**

Qian Liu,^a, #^ Kai Chen,^a, #^ Shaoshan Pan,^a^ Tao Zhang,^c^ Qian Wang,^a^ Jiaying Zhu,^a^ Jin Zhang,^c^ Xiaohua Jiang, ^a,*^ Haiying Jia,^c, *^ Shengxia Zheng,^a,*^ Tianzhi Luo_,_^a,*^

^a^ *Department of Modern Mechanics, Reproductive Medicine Center, The First Affiliated Hospital, State Key Laboratory of Fire Science, University of Science and Technology of China, Hefei, China*

^b^ *Jiangsu Hernrrie Biological and Medical Technology Company, Taizhou, China*

^c^ *The Ninth Medical Center of Chinese People’s Liberation Army General Hospital, Beijing, China*

**^#^** These authors contributed equally to this work

* Correspondence: X. Jiang at [biojxh@ustc.edu.cn](mailto:biojxh@ustc.edu.cn)

H. Jia at 1270802646@qq.com

S. Zheng at Sdl901zsx@163.com

T. Luo at tzluo@ustc.edu.cn

**Supplemental Data**

1. Supplementary Figures: S1-S16





Figure S1. Schematic diagram of mouse blastocyst extraction.

Uterine horns were dissected and the inner wall of the uterine horns were rinsed with M2 medium. The resulting solution contained blastocysts, morulae, granulosa cells, and some tissue fragments, which need to be repeatedly transferred in M2 medium to obtain pure blastocysts. Scale bar, 100 μm.





Figure S2. Quantification of embryonic morphology.

**(a**) Quantification and comparison of EPI height at different substrate stiffness levels. The 1kPa group was colored blue, the 4kPa group colored green, the 10kPa group colored red, and the 50kPa group colored orange. **(b)** Quantification and comparison of EPI width at different substrate stiffness levels. **(c)** Quantification and comparison of pTE height at different substrate stiffness levels. **(d)** Quantification and comparison of interface length at different substrate stiffness levels. **(e)** Quantification of interface diameter. Day2: 1kPa (n = 16), 4kPa (n = 16), 10kPa (n = 15), and 50kPa (n = 20). Day3: 1kPa (n = 12), 4kPa (n = 12), 10kPa (n = 13), and 50kPa (n = 22). Statistical analysis was performed using a two-tailed Student’s t-test. Scatterplot, mean ± SEM.





Figure S3. Immunostaining of embryonic morphology.

**(a**) Representative Z-stack images of embryo interacting with the 1 kPa substrate, with 4 μm intervals between optical sections. Green fluorescent beads labeling the hydrogel surface and embryos stained with F-actin (red). White arrows indicate regions where green fluorescent beads are out of the focal plane. Schematic illustrations are shown on the right. **(b**) Representative images of embryonic morphology on IVC day 2. Embryos stained with F-actin (green), phospho-Myosin Light Chain 2 (p-MLC, cyan), E-cadherin (E-cad, red), and nuclei (blue). Scale bar, 100 μm.





Figure S4. Nanoindentation for measuring the elastic modulus of mouse embryos.

**(a)** Brightfield image showed the elastic modulus measurement of mouse embryos vis nanoindentation. The small sphere marked in the image is the detection bead. Scale bar, 100 μm. **(b)** Stress-strain curves of embryonic ICM and TE at different substrate stiffness. The ICM was colored blue, the TE was colored red.



Figure S5. Two distinct embryonic morphologies.

Representative images of epiblast on day 3 of *in vitro* culture. The embryos stained with F-actin (green), E-cadherin (red) and nuclei (blue). For each group, the merged bright-field and fluorescence image is shown on top, with the corresponding fluorescence image below. A schematic diagram in the lower-right corner of each fluorescence image illustrates the corresponding structure.





Figure S6. Immunostaining of epiblast cytoskeleton.

**(a)** Representative images of epiblast on day 2 of *in vitro* culture. The embryos stained with F-actin (green), phospho-Myosin Light Chain 2 (cyan) and E-cadherin (red). **(b)** Merged plot profiles of the EPI and representative images of EPI on IVC day 3. Embryos stained with F-actin (green) and E-cadherin (red). **(c)** Representative images of epiblast on day 3 of *in vitro* culture. Red arrows highlighted regions of colocalization among actin, myosin, and E-cadherin. **(d)** Schematic diagram depicting the transition from a rosette structure to an EPI cavity during early embryogenesis. **(e)** Representative time-lapse images of actin-labeled embryonic rosettes treated with actin inhibitor (CytoD) or myosin II inhibitor (BLEB). Scale bar, 50 μm.


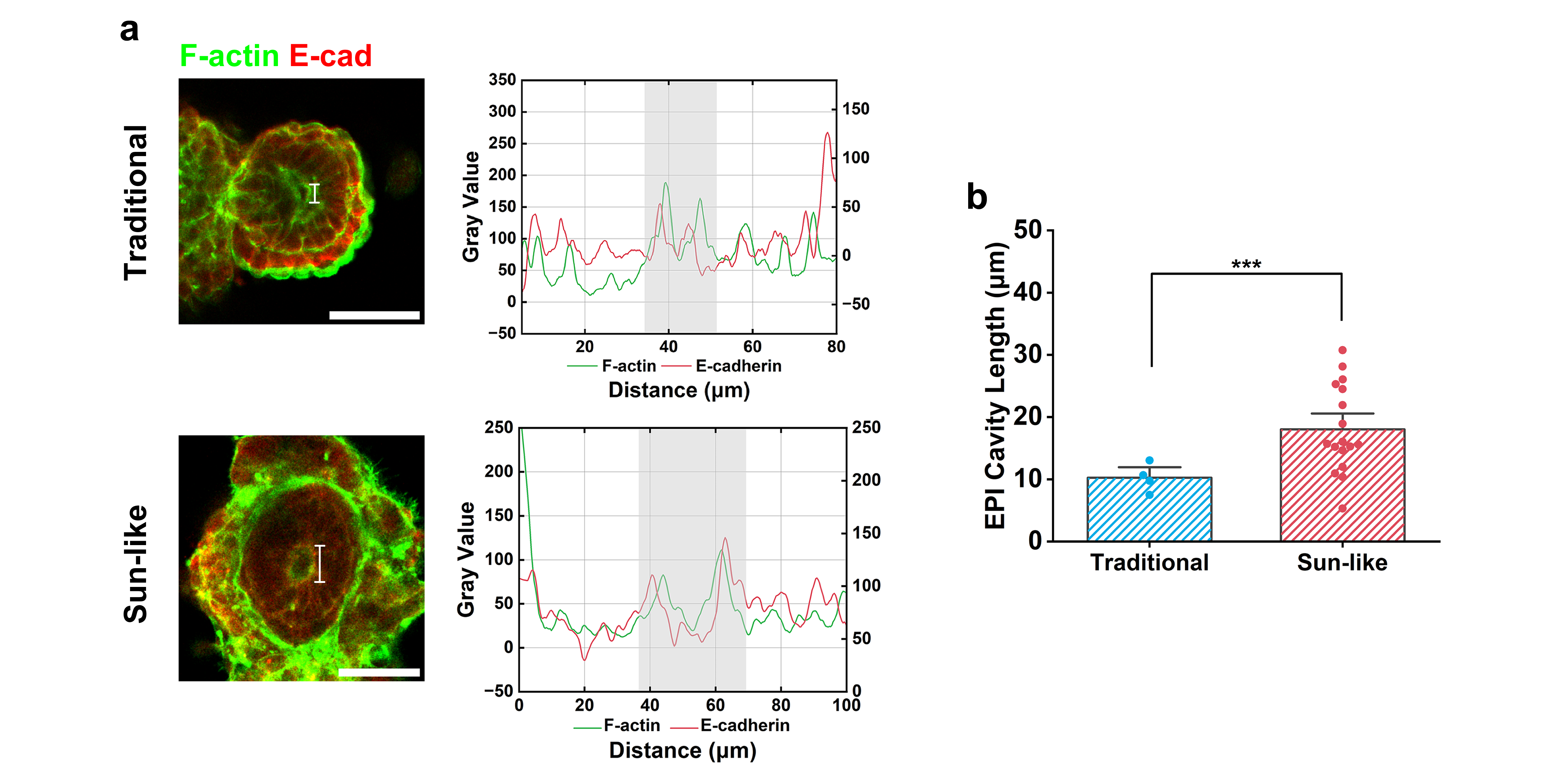
Figure S7. Two distinct assembly morphologies of embryos cultured on 1 kPa substrates.

**(a)** Representative images of the epiblast and merged plot profiles on day 3 of *in vitro* culture. The embryos stained with F-actin (green) and E-cadherin (red). The white line indicates the maximum length of the EPI cavity. Scale bar, 50 μm. **(b)** Quantification and comparison of EPI cavity length (μm) on IVC day 3. Traditional (n = 4), and Sun-like (n = 17).





Figure S8. Embryo adhesion processes.

**(a)** Time-lapse images of mouse embryo adhesion on substrates with different stiffness levels over 48 hours. Scale bar, 100 μm. **(b)** Changes in embryonic adhesion area and circularity over time. The time interval was 20 minutes. 1kPa (n = 11), 4kPa (n = 12), 10kPa (n = 12), and 50kPa (n = 11). Line graph, mean ± SEM.


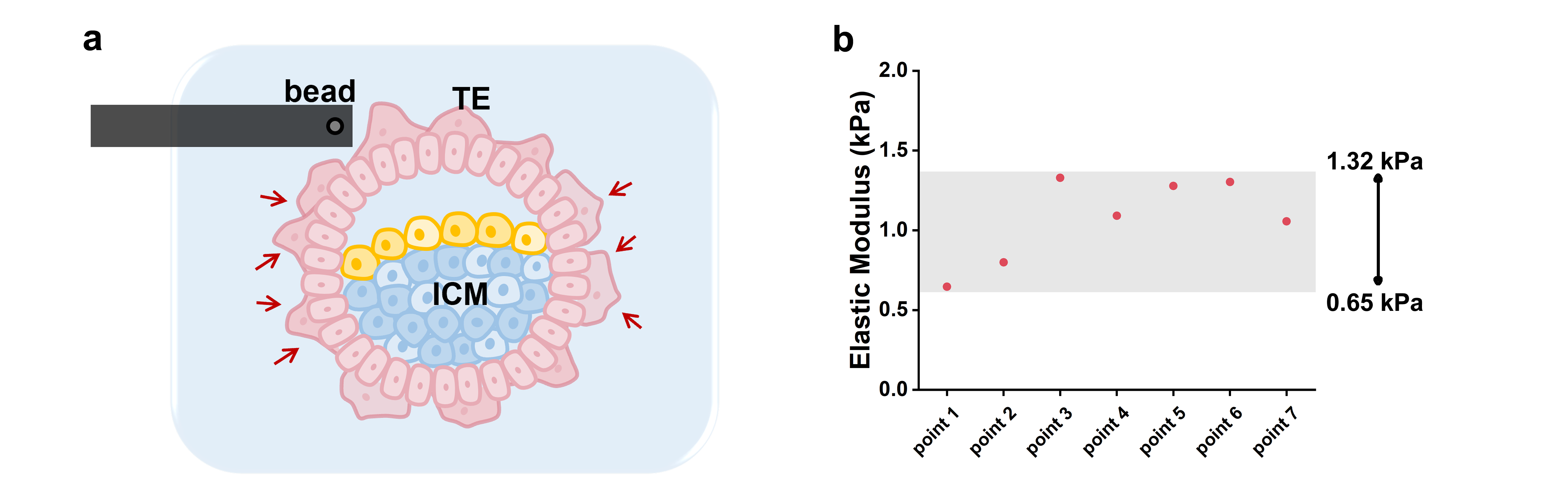
Figure S9. Measurement of substrate stiffness around attached embryos.

**(a)** Schematic of nanoindentation measurement of substrate stiffness surrounding the embryo following adhesion. **(b)** Distribution of elastic modulus at different locations around the embryo.





Figure S10. Dynamic monitoring of traction force during embryo adhesion on 1kPa substrates.

**(a)** Representative traction force field images of embryos adhering on 1kPa substrates. The images were captured over a period of 100 minutes. The time interval was 5 minutes. **(b)** Curve of max traction force (Pa) over time (n = 7). Dark blue shows representative data, while light blue indicates data from multiple replicates. **(c)** Curve of total traction force (nN) over time (n = 7). Dark red shows representative data, while light red indicates data from multiple replicates.





Figure S11. Migration data of embryos cultured on substrates with different stiffness.

**(a)** Representative migration tracking plots of embryos cultured on substrates with different stiffness levels over 12 hours. Different colors indicated data from multiple replicates. 1kPa (n = 16), 4kPa (n = 17), 10kPa (n = 15), and 50kPa (n = 14). **(b)** Migration speed varies over time, with a recording duration of 12 hours. 1kPa (n = 16), 4kPa (n = 17), 10kPa (n = 15), and 50kPa (n = 14). **(c)** Migration speed of embryos cultured substrates with different stiffness levels. Mean speed was calculated by dividing the total migration distance (μm) by the overall duration (min). 1kPa (n = 16), 4kPa (n = 17), 10kPa (n = 14), and 50kPa (n = 14). **(d)** Directionality ratio of embryos cultured on substrates with different stiffness levels. 1kPa (n = 16), 4kPa (n = 17), 10kPa (n = 15), and 50kPa (n = 14). **(e)** Mean square displacement (MSD) and time intervals are shown in a log-log plot. 1kPa (n = 15), 4kPa (n = 14), 10kPa (n = 15), and 50kPa (n = 15). Line graph and scatterplot, mean ± SEM.





Figure S12. Dynamic monitoring of traction force during embryo adhesion on 4kPa substrates.

**(a)** Representative traction force field images of embryos adhesion on 4kPa substrates. The images were captured over a period of 4 hours. **(b)** Curve of max traction force (Pa) and total traction force (nN) over time. The max traction force is indicated by the blue line, while the total traction force is represented by the red line. **(c)** Representative traction force field images (top panel) and bright field images (bottom panel) of embryos. The images were captured over a period of 4 hours. **(d)** Correlation analysis of max traction force and displacement during embryonic development on 4kPa substrates. The max traction force is indicated by the blue line, while the displacement is represented by the red line. **(e)** Frequency analysis of periodic max traction force. Data were from 13 embryos across 2 independent experiments.





Figure S13. Representative confocal Z section images of embryos cultured on 1kPa substrates.

Embryos stained with the EPI marker OCT4 (green) and extraembryonic ectoderm marker CDX2 (red). Z-slice images were arranged in order from low to high, with a 2 μm interval. Scale bar, 100 μm.



Figure S14. Traction force analysis of embryos treated with YAP-related inhibitors.

**(a)** Percentage of attached embryos treated with YAP-related inhibitors on IVC day 2 (n = 50). The DMSO treated group served as the control, while the experimental groups included treatment with the YAP nuclear export inhibitor (Leptomycin B) and YAP inhibitor (Dasatinib and Verteporfin). **(b)** Quantification of max traction force (Pa) of embryos treated with different inhibitors. DMSO (n = 47), Leptomycin B (n = 47), Dasatinib (n = 47), and Verteporfin (n = 47). **(c)** Quantification of total traction force (nN) of embryos treated with different inhibitors. DMSO (n = 42), Leptomycin B (n = 39), Dasatinib (n = 37), and Verteporfin (n = 36). **(d)** Representative traction force field images of embryos treated with YAP-related inhibitors. **(e)** Curves showing the max traction force (Pa, left) and total traction force (nN, middle) over time following YAP-related inhibitor treatment, and the frequency analysis of periodic max traction forces (right). LMB (n = 18) and VP (n = 17). Statistical analysis was performed using a two-tailed Student’s t-test. Scatterplot, mean ± SEM.





Figure S15. Clustering heatmap of differentially expressed genes in embryos treated with YAP-related inhibitors.

**(a)** The differentially expressed gene set was generated by taking the union of differentially expressed genes across all comparison groups. The x-axis represents sample names. The y-axis represents the FPKM-normalized values of differentially expressed genes. Red intensity indicates higher expression levels, while blue intensity indicates lower expression levels. **(b)** Scaled expression heatmap of specific marker genes related to hematopoiesis (i) and organogenesis (ii), based on clustering analysis of RNA-seq data.





Figure S16. Differential expression analysis of cytoskeletal and extracellular matrix proteins.

**(a-b)** GO enrichment analysis of cytoskeletal and extracellular matrix-related terms following treatment with YAP-related inhibitors. Categories without significant differences are highlighted in gray. **(c)** Cytoskeleton-related genes further filtered from the gene set, including those for actin, microtubules, intermediate filaments, and myosin. **(d)** Extracellular matrix (ECM)-related genes, including those for collagen, integrins, focal adhesions, and matrix metalloproteinases.
